# Simvastatin Ameliorates PAK4 Inhibitor-Induced Gut and Lung Injury

**DOI:** 10.1155/2017/8314276

**Published:** 2017-12-28

**Authors:** Shuming Pan, Zengbin Wu, Xuan Liu, Jiameng Chen, Huiqi Wang, Dan Liu, Aihua Fei, Liang Chen, Chengjin Gao

**Affiliations:** ^1^Emergency Department, Xinhua Hospital Affiliated to Shanghai Jiao Tong University School of Medicine, Shanghai 200092, China; ^2^Emergency Department, Shanghai Tenth People's Hospital, Shanghai 200072, China

## Abstract

P21 activated kinase 4 (PAK4), a key regulator of cytoskeletal rearrangement and endothelial microparticles (EMPs), is released after lipopolysaccharide (LPS) stimulation. In addition, it participates in LPS-induced lung injury. In this study, forty-eight Sprague Dawley (SD) rats were divided into two groups, including PAK4 inhibitor (P) and PAK4 inhibitor + simvastatin (P + S) treatment groups. All rats were given PAK4 inhibitor (15 mg/kg/d) orally. Immediately after PAK4 inhibitor administration, simvastatin was injected intraperitoneally to P + S group animals at 20 mg/kg/day. Then, treatment effects on the intestinal mucosal barrier and lung injury caused by PAK4 inhibitor and simvastatin were assessed. The results showed that gut Zonula Occludens- (ZO-) 1, PAK4, mitogen-activated protein kinase 4 (MPAK4), and CD11c protein levels were reduced, while plasma endotoxin levels were increased after administration of PAK4 inhibitor. Furthermore, compared with normal rats, wet-to-dry (W/D) values of lung tissues and circulating EMP levels were increased in the treatment group, while PAK4 and CD11c protein amounts were reduced. Therefore, in this lung injury process induced by PAK4 inhibitor, the protective effects of simvastatin were reflected by intestinal mucosal barrier protection, inflammatory response regulation via CD11c+ cells, and cytoskeleton stabilization. In summary, PAK4 is a key regulator in the pathophysiological process of acute lung injury (ALI) and can be a useful target for ALI treatment.

## 1. Introduction

Sepsis is one of the principal causes of death in critical care medicine departments [1]. Despite the continuous development of modern treatment methods, the overall mortality rate of sepsis remains high and is tightly associated with disease severity [2]. According to SEPSIS 3.0, organ dysfunction is the main characteristic of septic patients, who commonly develop lung failure. Although acute lung injury (ALI) can result from multiple intrapulmonary and extrapulmonary pathological stimuli [3], sepsis remains the leading risk factor. As plasma endotoxin is a pivotal factor in sepsis-induced lung injury, lipopolysaccharide (LPS) injection and cecal-ligation/puncture (CLP) models are used in multiple basic studies to explore the pathophysiological mechanisms of sepsis-induced ALI [4, 5]. In critically ill patients, gut barrier dysfunction is very common and considered a component of distant organ failure, including lung injury [6]. The gut and its contents act as a reservoir for bacteria and endotoxins, which can subsequently induce systemic inflammatory response syndrome (SIRS), ALI, and multiple organ dysfunction syndrome (MODS) [7]. Gut barrier dysfunction is one of the main factors in this pathological process, which is considered a loss of intestinal mucosal integrity, leading to bacterial translocation and the release of gut-derived proinflammatory cytokines [8].

ALI is associated with pulmonary hypervascular permeability, edema, and high levels of proinflammatory cytokines [9, 10]. Disruption of the vascular endothelial barrier results in ALI, which is a consequence of cytoskeletal rearrangement in pulmonary endothelial cells [11]. We previously demonstrated that enhanced circulating endothelial microparticle (EMP) levels [4, 12] result from disorders of the pulmonary vascular endothelial cytoskeleton. EMP release is regulated by p21 activated kinase 4 (PAK4), an effector of Rho GTPases, which alter the cell cytoskeleton. PAKs are well-known effector proteins for the Rho GTPases Cdc42 and Rac. As a serine/threonine kinase, PAK4 is a type II PAK that specifically binds strongly to Cdc42 [13], which was originally described as a regulator of cytoskeletal dynamics and cell motility. Thanks to its regulatory effects on the cell cytoskeleton, PAK4 is considered a potential target for assessing the pathophysiological process of ALI [14]; this would help develop optimal treatments. A previous study demonstrated that PAK4 inhibitors cause intestinal mucosa injury [15]. We previously demonstrated that simvastatin attenuates LPS-induced ALI via cytoskeleton stabilization by regulating the pulmonary Cdc42-PAK4 pathway and altering the levels of circulating endothelial microparticles (EMPs) [4]. PAK4 is a promising factor involved in the dysfunction of the gut mucosa barrier and pulmonary vascular endothelial cytoskeleton; however, to unveil the underlying mechanisms, further studies are required.

In the current study, rats were orally given the PAK4 inhibitor PF-3758309. First, the effects of the PAK4 inhibitor on gut barrier function were assessed, quantifying the protein levels of PAK4, ZO-1, CD11c, and MAP4K4. Meanwhile, a relationship between the effect of the PAK4 inhibitor and the degree of lung injury was determined by plasma endotoxin levels, EMP amounts in circulation, PAK4 and CD11c protein levels, and histological alterations of the lung. Finally, the protective effects of simvastatin on PAK4 inhibitor-induced gut and lung dysfunction were also evaluated.

## 2. Materials and Methods

### 2.1. Animal Model

Seven-week-old male Sprague Dawley (SD) rats (250 ± 10 g) were used in this study. All experimental protocols were approved by the Institutional Animal Care and Use Committee of Shanghai Jiao Tong University and performed in strict accordance with the National Institutes of Health guidelines for the use of experimental animals.

### 2.2. Experimental Design

Forty-eight animals were randomly divided into two groups (*n* = 24), respectively, named PAK4 inhibitor (P) and PAK4 inhibitor + simvastatin (P + S) treatment groups. The PAK4 inhibitor was administered orally once daily at a dose of 15 mg/kg [16] to all experimental animals. Immediately thereafter, simvastatin was given intraperitoneally to the P + S group at 20 mg/kg/day. Second and third administrations (PAK4 inhibitor, PAK4 inhibitor + simvastatin) were performed at 24 and 48 h, respectively, after the initial injection. Animals were sacrificed at 24, 48, and 72 h after the initial injection, respectively, by exsanguination via cardiac puncture. Eight normal rats constituted the control group and were intraperitoneally injected with the same volume of normal saline. Another eight normal rats were intraperitoneally given simvastatin at 20 mg/kg and sacrificed at 24 h after administration. Then, the protein expression levels of PAK4 and CD11c in lung tissues from the rats were assessed.

### 2.3. Antibodies

We used specific antibodies raised against PAK4 (Santa Cruz Biotechnology, CA, USA), ZO-1 (Life Technologies Corporation, Carlsbad CA, USA), CD11c (Novocastra, Newcastle upon Tyne, UK), and MAP4K4 (Cell Signaling Technology Inc., Massachusetts, USA).

### 2.4. Pulmonary Function Assessed by Lung Wet-to-Dry (W/D) Ratio and Alveolar Wall Thickness Measurements

At 24, 48, and 72 h after administration of PAK4 inhibitor or PAK4 inhibitor + simvastatin, the animals were exsanguinated by cardiac puncture. Then, the two lungs were harvested from each animal and separated: the right lung was used for subsequent tests (histological examination and Western blot), while the left one was homogenized and weighed. Afterward, the homogenate was centrifuged (14,000 ×g, 10 min) and desiccated in an oven (70°C for 24 h) for dry weight determination. Lung wet-to-dry weight ratio (W/D) was then derived. Meanwhile, hematoxylin-eosin (H&E) staining was performed for all specimens. Six high power fields were randomly selected in every section, and the Spot Advanced Computer Photo Analysis Microsoft System (Silicon Graphic Inc., USA) was used to measure alveolar wall thickness.

### 2.5. Immunofluorescence and Confocal Microscopy

Gut and lung tissue specimens from each rat were obtained after perfusion with cold PBS and 2% paraformaldehyde. After two hours of fixation with 2% paraformaldehyde, the samples were cryopreserved for later use. Gut and lung sections (6 *μ*m) were permeabilized with 0.2% Triton X-100 for 20 minutes and stained as described previously [16]. The protein levels of PAK4, ZO-1, CD11c, and MAP4K4 were determined by using the respective antibodies. Images in six random fields/sections were acquired under a confocal fluorescence microscope (Axiovert 100, LSM 510, Zeiss, Germany).

### 2.6. EMP Analyses

Five hundred microliters of blood were obtained from each rat. Circulating EMPs were isolated as previously described [17]. Blood cells were removed by 2 centrifugations at 2,500 ×g for 15 minutes, and platelet-free plasma (PFP) was obtained. Blood EMPs were measured by flow cytometry (BD FACSCanto II; BD Biosciences, San Jose, CA) as previously described [18, 19].

### 2.7. Western Blot

Gut and lung tissues were perfused with Hanks' balanced solution, hand minced, mixed with 1% sodium dodecyl sulfate (SDS) in PBS containing protease inhibitors, and homogenized (3 × 30 s on ice) on a Polytron (Westbury, NY). Tissue lysates were cleared by centrifugation (14,000 ×g, 30 min) on a Beckman Avanti Centrifuge J-25 I using a JA-25.50 rotor. The subsequent procedures were based on our previous study [20]. Primary antibodies were raised against PAK4 (1 : 1000), ZO-1 (1 : 1000), CD11c (1 : 1000), and MAP4K4 (1 : 1000).

### 2.8. Determination of Endotoxin Protein Content

The protein content of endotoxins was detected by DC Protein Assay (Bio-Rad Laboratories, Hercules, CA).

### 2.9. Statistical Analysis

Data are mean ± standard error (SE) and were statistically analyzed with the SPSS version 16.0 software (SPSS Inc., USA). Student-Newman-Keuls-*q* test, Student's *t*-test (between two groups), and two-way analysis of variance (ANOVA) (>2 groups) were used for comparisons. *P* < 0.05 was considered statistically significant.

## 3. Results

### 3.1. Effects of PAK4 Inhibitor and Simvastatin on Lung Tissues

**Figure 1:**
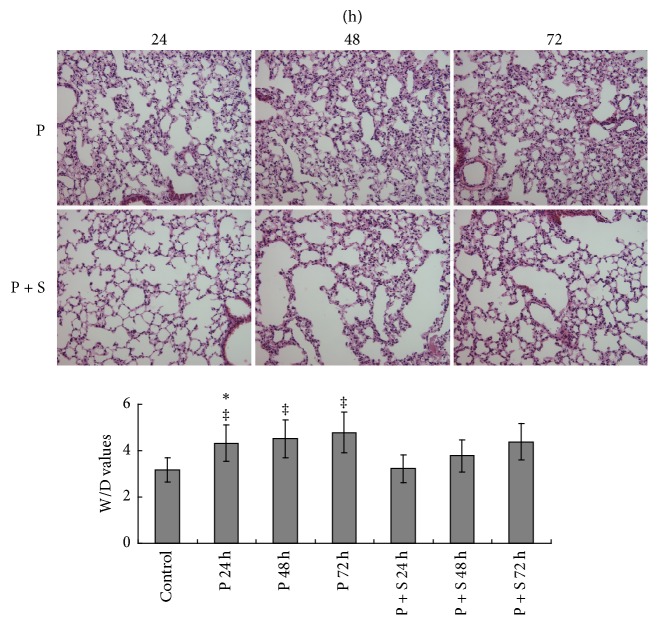
Effect of the PAK4 inhibitor and simvastatin on lung tissues. The alveolar wall was thickened, and wet-to-dry (W/D) values were increased after administration of the PAK4 inhibitor; meanwhile, simvastatin alleviated these effects. ^‡^*P* < 0.05 versus control rats; ^*∗*^*P* < 0.05 versus P + S treated rats.

As shown in Figure 1, the PAK4 inhibitor induced alveolar wall thickening and increased W/D values. At 24, 48, and 72 h after administration, mean alveolar wall thicknesses in the rats were 2.11 ± 0.37, 2.67 ± 0.44, and 2.79 ± 0.53 *μ*m, respectively, in the P group, and 1.39 ± 0.18, 1.85 ± 0.31, and 2.05 ± 0.37 *μ*m, respectively, in the P + S group; mean alveolar wall thickness was 0.89 ± 0.04 *μ*m in normal rats. Significant differences between the P and P + S groups at 24, 48, and 72 h (all *P* < 0.05) were found. W/D values were greatly increased in the lungs of PAK4 inhibitor-treated rats but remained low in those given simvastatin.

### 3.2. Effects of PAK4 Inhibitor and Simvastatin on EMPs

**Figure 2:**
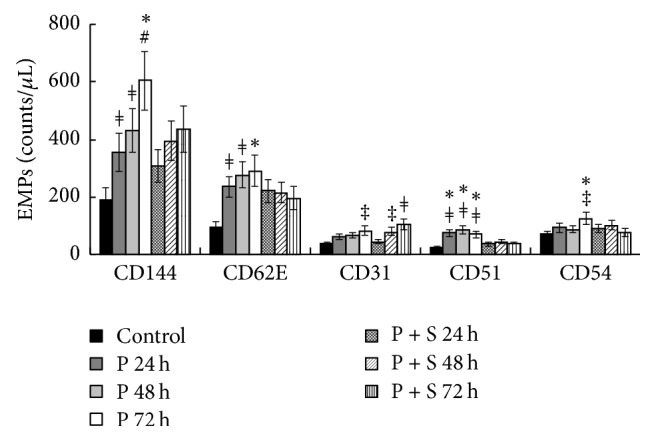
The PAK4 inhibitor induces endothelial microparticle (EMP) release* in vivo*, an effect alleviated by simvastatin. ^#^*P* < 0.001 versus control rats; ^*ǂ*^*P* < 0.01 versus control rats; ^‡^*P* < 0.05 versus control rats; ^*∗*^*P* < 0.05 versus P + S treated rats.

EMPs with different phenotypes were counted (Figure 2). Following PAK4 inhibitor administration, the amounts of plasma CD144+, CD62E+, CD31+, CD51+, and CD54+ EMPs were obviously increased at different time-points (*P* < 0.01 for CD144 at 24–48 h, *P* < 0.001 for CD144 at 72 h, *P* < 0.01 for CD62E at 24–72 h, *P* < 0.05 for CD31 at 72 h, *P* < 0.01 for CD51 at 24–72 h, and *P* < 0.05 for CD54 at 72 h, versus control, resp.).

However, compared with the P group, the P + S group showed reduced EMP amplification (*P* < 0.05 for CD144 at 72 h, *P* < 0.05 for CD62E at 72 h, *P* < 0.05 for CD31 at 24–72 h, *P* < 0.05 for CD51 at 24–72 h, and P< 0.05 for CD54 at 72 h, resp.).

### 3.3. Effects of the PAK4 Inhibitor and Simvastatin on Gut PAK4, CD11c, ZO-1, and MAP4K4 Levels

**Figure 3:**
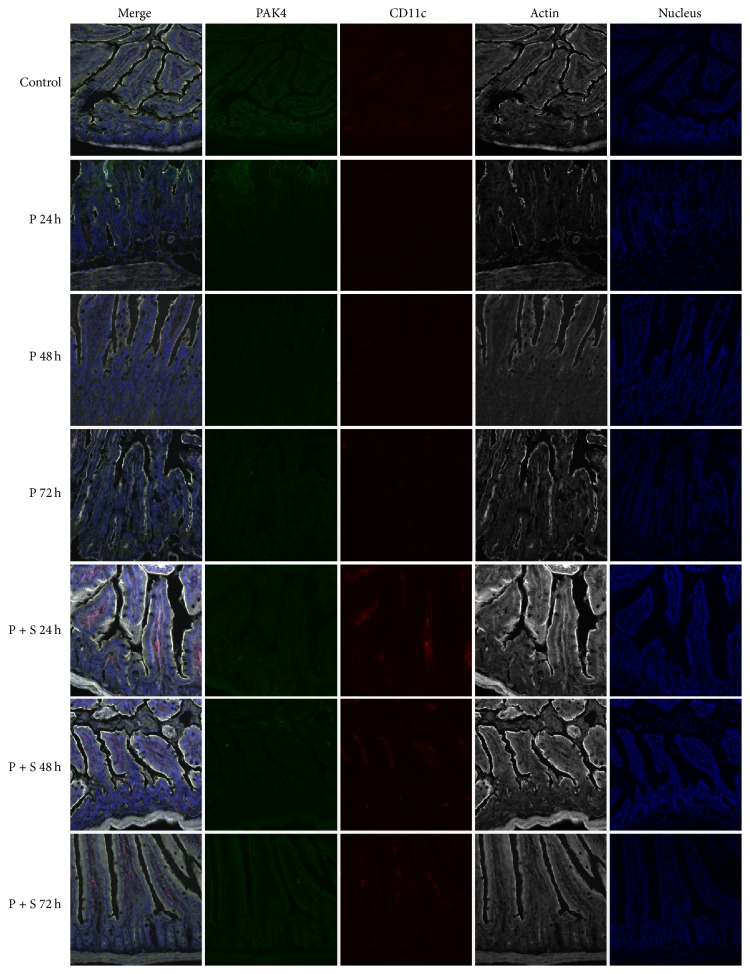
Immunofluorescent images of p21 activated kinase 4 (PAK4) and CD11c detection in gut tissues. Significant differences were found between control animals and the PAK4 inhibitor-treatment group (all *P* < 0.01), as well as between the P and P + S groups at 48 and 72 h (*P* < 0.05 and *P* < 0.01, resp.). Green, PAK4; red, CD11c; white, actin.

The expression levels of PAK4 and CD11c in gut tissues from all animals were assessed, and fluorescence intensities are shown in Figure 3. The relative protein levels of PAK4 were 13.52 ± 2.37 (Control), 8.61 ± 1.47 (P; 24 h), 7.19 ± 1.55 (P; 48 h), 7.83 ± 1.65 (P; 72 h), 10.67 ± 2.17 (P + S; 24 h) 11.34 ± 2.58 (P + S; 48 h), and 13.95 ± 2.58 (P + S; 72 h). There were significant differences between control animals and those treated with the PAK4 inhibitor (all *P* < 0.01, resp.). Furthermore, significant differences were found between the P and P + S groups at 48 and 72 h, respectively (*P* < 0.05 and *P* < 0.01, resp.).

**Figure 4:**
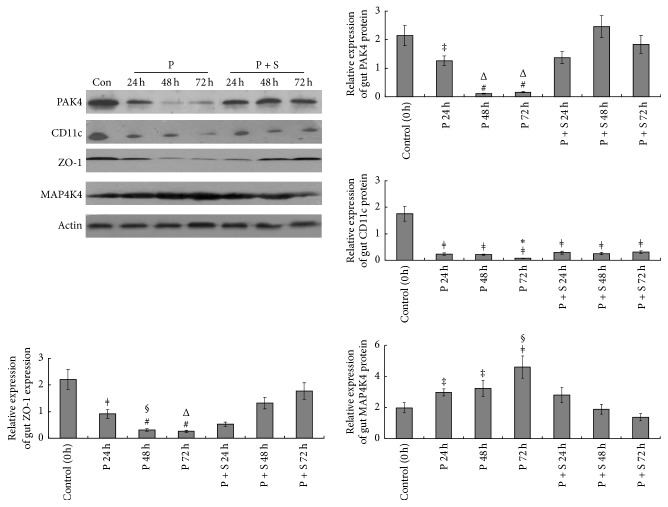
Western blot analysis of p21 activated kinase 4 (PAK4), CD11c, ZO-1, and MAP4K4 in gut tissues. Protein levels were normalized to *β*-actin. ^#^*P* < 0.001 versus control rats; ^*ǂ*^*P* < 0.01 versus control rats; ^‡^*P* < 0.05 versus control rats; ^*∗*^*P* < 0.05 versus P + S treated rats; ^§^*P* < 0.01 versus P + S treated rats; ^Δ^*P* < 0.001 versus P + S treated rats.

Relative CD11c protein expression levels in gut tissues were 3.72 ± 0.64 (Control), 0.49 ± 0.07 (P; 24 h), 0.36 ± 0.05 (P; 48 h), 0.58 ± 0.09 (P; 72 h), 4.15 ± 0.83 (P + S; 24 h), 3.47 ± 0.68 (P + S; 48 h), and 4.34 ± 0.95 (P + S; 72 h), indicating significant differences between control animals and those given the PAK4 inhibitor (all *P* < 0.001). What is more, significant differences were found between the P and P + S groups at the same time-point (all *P* < 0.001). Western blot (Figure 4) showed the same tendency for PAK4 and CD11c expression levels in gut tissues.

We also detected the protein levels of ZO-1 and MAP4K4 in gut tissues by Western blot (Figure 4). Interestingly, the PAK4 inhibitor significantly decreased ZO-1 protein levels at 24, 48, and 72 h (*P* < 0.01, *P* < 0.001, and *P* < 0.001 versus control, resp.), while decreasing MAP4K4 protein amounts (all *P* < 0.05 versus control). However, the protein levels of ZO-1 were much higher after simvastatin administration at 48 and 72 h (*P* < 0.05 and *P* < 0.01, resp.) compared with P group amounts. Meanwhile, MAP4K4 protein levels were much higher after simvastatin administration at 24, 48, and 72 h (all *P* < 0.05).

### 3.4. Effects of the PAK4 Inhibitor and Simvastatin on Lung PAK4 and CD11c Amounts

**Figure 5:**
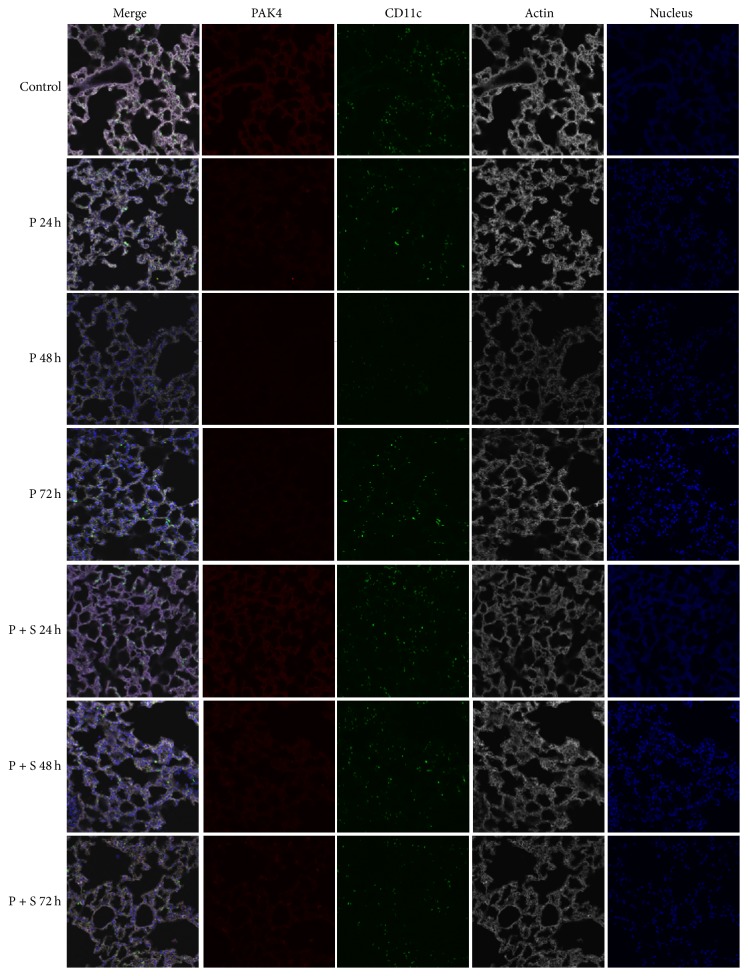
Immunofluorescent images of p21 activated kinase 4 (PAK4) and CD11c detection in lung tissues. Green, PAK4; red, CD11c; white, actin.

Figure 5 shows fluorescence intensities of the PAK4 and CD11c proteins in lung tissues. Relative expression levels of the PAK4 protein were 6.72 ± 1.23 (Control), 6.35 ± 1.14 (S; 24 h), 2.39 ± 0.47 (P; 24 h), 1.46 ± 0.31 (P; 48 h), 1.67 ± 0.44 (P; 72 h), 5.46 ± 1.02 (P + S; 24 h), 5.68 ± 1.12 (P + S; 48 h), and 4.37 ± 0.81 (P + S; 72 h). These findings indicated significant differences between control animals and the PAK4 inhibitor group (all *P* < 0.01). Furthermore, significant differences between the P and P + S groups were obtained at 24, 48, and 72 h (all *P* < 0.05); however, there was no obvious difference between the control and simvastatin groups at 24 h (S; 24 h).

Relative expression levels of the CD11c protein in lung tissues were 7.51 ± 1.36 (Control), 7.86 ± 1.59 (S; 24 h), 5.92 ± 0.97 (P; 24 h), 2.36 ± 0.41 (P; 48 h), 4.37 ± 0.93 (P; 72 h), 5.37 ± 0.91 (P + S; 24 h), 5.21 ± 1.03 (P + S; 48 h), and 4.34 ± 0.87 (P + S; 72 h), indicating marked differences between control animals and the PAK4 inhibitor-treatment group, particularly at 48 and 72 h (*P* < 0.01 and *P* < 0.05, resp.). There was also an overt difference between the P and P + S groups at 48 h (*P* < 0.01), with no significant difference between the control and simvastatin groups at 24 h (S; 24 h).

**Figure 6:**
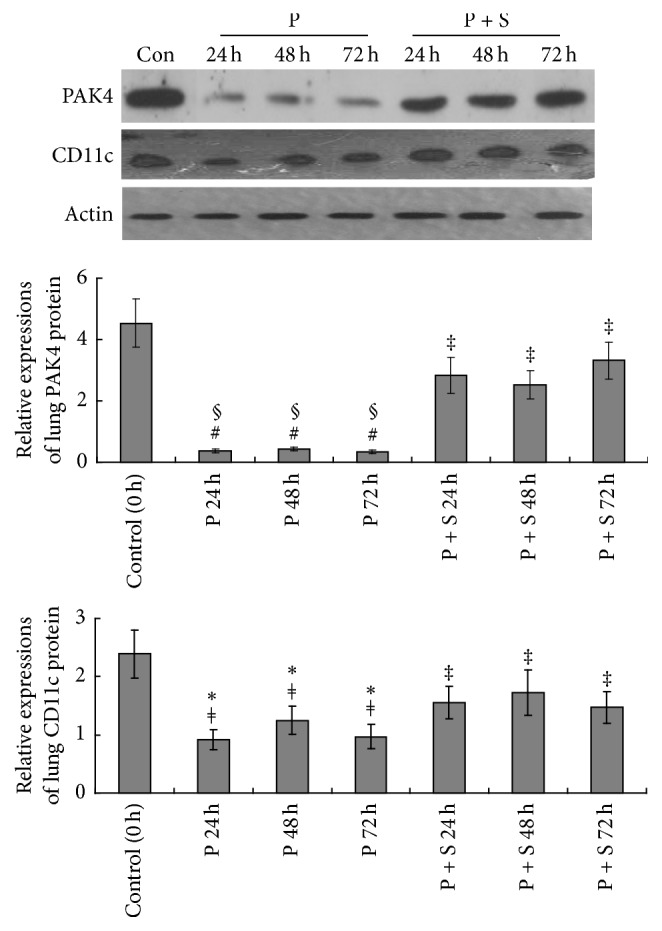
Western blot analysis of p21 activated kinase 4 (PAK4) and CD11c detection in lung tissues. Protein levels were normalized to *β*-actin. ^#^*P* < 0.001 versus control rats; ^*ǂ*^*P* < 0.01 versus control rats; ^‡^*P* < 0.05 versus control rats; ^*∗*^*P* < 0.05 versus P + S treated rats; ^§^*P* < 0.01 versus P + S treated rats.

Western blot (Figure 6) directly indicated that the PAK4 inhibitor significantly decreased PAK4 protein levels in lung tissues at 24, 48, and 72 h (all *P* < 0.001, resp.). In addition, PAK4 protein levels were much higher than those of the P group after simvastatin treatment at 24, 48, and 72 h (all *P* < 0.01). CD11c protein levels were also strikingly decreased after PAK4 inhibitor treatment at 24, 48, and 72 (all *P* < 0.01). Compared with P group amounts, CD11c protein levels were much higher after simvastatin treatment at 24, 48, and 72 h (all *P* < 0.05).

### 3.5. Effects of the PAK4 Inhibitor and Simvastatin on Plasma Endotoxin Levels

**Figure 7:**
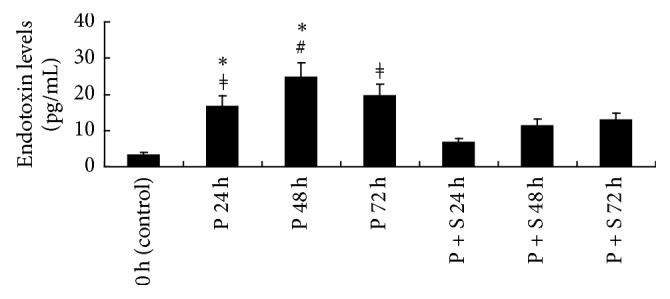
Plasma endotoxin levels in both groups. ^#^*P* < 0.001 versus control rats; ^*ǂ*^*P* < 0.01 versus control rats; ^*∗*^*P* < 0.05 versus P + S treated rats.

Figure 7 shows plasma endotoxin levels for all experimental animals. After PAK4 inhibitor injection, plasma endotoxin levels in rats were enhanced and peaked at 48 h. Endotoxin levels in the P group were all higher than control values (*P* < 0.01 at 24 h, *P* < 0.001 at 48 h, and *P* < 0.01 at 72 h) and P + S treated animals at 24 and 48 h (all *P* < 0.05).

## 4. Discussion

Despite significant investigational efforts, abdominal sepsis is a major cause of mortality in ICU [21]. The intestine is considered one of the largest immune barrier systems, and the main source of bacteria and endotoxins in the body [22]. Dysfunction of the intestinal mucosal barrier directly triggers mucosal hyperpermeability and gut bacterial translocation, both of which subsequently lead to local intestine damage or distant lung injury by inducing systemic inflammatory response syndrome (SIRS), which is associated with organ damage. Meanwhile, the lung is the most vulnerable and critical target organ in patients with sepsis. Approximately 40% of sepsis patients develop ALI, making sepsis the most common cause of ALI [23]. Increased pulmonary microvascular permeability contributes to respiratory failure in ALI; PAK4-associated pulmonary vascular cytoskeletal rearrangement is also involved in increased vascular permeability, alveolar edema, and LPS-induced ALI [4]. Furthermore, recent findings suggest that PAK4 has a close relationship with the intestinal mucosal barrier, as shown by PAK4 inhibitor associated damage [15].

In this study, an animal model of oral PAK4 inhibitor was established to assess the relationship between the intestinal mucosal barrier and pulmonary vascular cytoskeleton dysfunction. After oral administration of 15 mg/kg/d PAK4 inhibitor to rats, gut PAK4 and ZO-1 protein levels were significantly decreased. ZO-1 is a key component of tight junctions, which seal the paracellular space between adjacent intestinal epithelial cells [24]. Accumulating evidence suggests that tight-junction opening can be triggered by cytoskeletal rearrangement. After treatment with the PAK4 inhibitor, gut protein levels of MAP4K4 in rats were increased. As a member of the sterile-20 protein kinase family, MAP4K4 is closely related to the protein kinases Misshapen-like kinase 1 (MINK1), Traf2, and Nik-interacting kinase (TNIK), which are important in both cytoskeletal rearrangement and tight-junction permeability [25]. Alterations of gut PAK4, ZO-1, and MAP4K4 levels by the PAK4 inhibitor confirmed the relationship between cytoskeletal regulators and tight-junction components.

Moreover, MAP4K4 also plays a key regulatory role in inflammation, emerging as a new target for the suppression of LPS-induced macrophage inflammatory responses. In the present study, MAP4K4 levels in gut and lung tissues were increased after administration of the PAK4 inhibitor, suggesting enhanced MAP4K4-associated inflammatory response [26, 27]. Furthermore, the possible immune mechanisms of gut and lung tissues after treatment with the PAK4 inhibitor were explored, and there were less CD11+ cells, which are integral members of mammalian innate and adaptive immune responses, in comparison with control values [28]. In mouse, dendritic cell (DC) subsets and some macrophage populations express high levels of CD11c; indeed, CD11c+ macrophages and DCs are the main pathogen-sensing cells of the innate immune system in the lung. Depletion of CD11c+ cells renders rats dramatically more susceptible to progression from ALI to systemic distress and death [29]. The correlation between CD11c suppression and lung injury in this study strongly supported previous findings that CD11c+ cells play an important regulatory role in the pathophysiological process of ALI.

PAK4 inhibitors, as toxic agents to the gastrointestinal tract, inhibit PAK4 expression and cause gut intestinal mucosa epithelial cell injury. Alterations of ZO-1, PAK4, MAP4K4, and CD11c in gut tissues suggested gut injury, and dysfunction of gut intestinal mucosa epithelial cells directly leads to loss of mucosal integrity and favors bacterial translocation. The increased plasma endotoxin levels after administration of the PAK4 inhibitor also proved loss of mucosal integrity and bacterial translocation.

Multiple studies have suggested mechanisms for endotoxin- (LPS-) induced lung injury. We also found that PAK4, which is associated with increased plasma EMP levels, could constitute a useful pulmonary injury marker and an effective therapeutic target in LPS-treated mice [4]. As shown above, alveolar wall thickening and higher W/D values of lung tissues were obtained, coupled with decreased pulmonary PAK4 and increased circulating EMP levels after administration of the PAK4 inhibitor. These results rightly confirmed our previous findings of PAK4-associated EMP release in LPS-induced lung injury. In this study, lung injury originated from pulmonary cytoskeletal rearrangement and plasma endotoxin associated dysfunction of the intestinal mucosal barrier, both of which are caused by the PAK4 inhibitor.

As mentioned above, PAK4 is a key regulator of cytoskeletal rearrangement and EMP release and can be considered a potential target for ALI treatment. In addition, simvastatin has been used in our previous and present studies to alleviate pulmonary injury by enhancing PAK4 expression, reducing PAK4-associated cytoskeletal rearrangement, and decreasing EMP release [4]. We previously found that PAK4 participates in both LPS-induced and ventilation induced lung injury by regulating EMP release [4, 19], while simvastatin shows protective effects against lung injury [4]. In the present study, the protective effects of simvastatin against lung injury were also elucidated, as reported previously. Stabilizing the cell cytoskeleton and tight junctions is the main mechanism by which simvastatin alleviates lung/gut injury. However, simvastatin (20 mg/kg, 24 h) showed no effects on pulmonary PAK4 and CD11c expression levels in normal rats. Furthermore, simvastatin doses in this and other studies were indeed much higher than the physiological value, and muscle toxicity could not be evaluated. Therefore, the present findings should be expanded to other animal models, and further studies assessing the clinical usefulness of simvastatin in ALI are warranted. This study confirmed the potential role of PAK4 as a key regulator in the pathophysiological process of ALI, offering a useful treatment target for this ailment.
